# Identification of the amino acid residue responsible for the myricetin sensitivity of human proton-coupled folate transporter

**DOI:** 10.1038/s41598-019-54367-9

**Published:** 2019-12-02

**Authors:** Takahiro Yamashiro, Tomoya Yasujima, Kinya Ohta, Katsuhisa Inoue, Hiroaki Yuasa

**Affiliations:** 1 Department of Biopharmaceutics, Graduate School of Pharmaceutical Sciences, Nagoya City University, Nagoya, Japan; 2 College of Pharmacy, Kinjo Gakuin University, Nagoya, Japan; 3 Department of Biopharmaceutics, School of Pharmacy, Tokyo University of Pharmacy and Life Sciences, Tokyo, Japan

**Keywords:** Membrane proteins, Transporters

## Abstract

Human proton-coupled folate transporter (hPCFT/SLC46A1) has recently been found to be inhibited by myricetin by a sustained mechanism, raising a concern that the inhibition might lead to malabsorption of folates in the intestine, where hPCFT works for their epithelial uptake. However, rat PCFT (rPCFT) has more recently been found not to be inhibited by myricetin. Prompted by this finding, we attempted to determine the amino acid residue involved in that by analyses comparing between hPCFT and rPCFT. In the initial analysis, chimeric constructs prepared from hPCFT and rPCFT were examined for myricetin sensitivity to determine the hPCFT segment involved in the sensitivity. Focusing on the thereby determined segment from 83rd to 186th amino acid residue, hPCFT mutants having a designated amino acid residue replaced with its counterpart in rPCFT were prepared for the subsequent analysis. Among them, only G158N-substituted hPCFT was found to be transformed to be insensitive to myricetin and, accordingly, oppositely N158G-substituted rPCFT was transformed to be sensitive to myricetin. These results indicate the critical role of Gly^158^ in the myricetin sensitivity of hPCFT. This finding would help advance the elucidation of the mechanism of the myricetin-induced inhibition of hPCFT and manage the potential risk arising from that.

## Introduction

Human proton-coupled folate transporter (hPCFT/SLC46A1) was identified about a decade ago as the transporter that operates for the epithelial uptake of folate (vitamin B9) and analogues in the intestine^1^. Since then, studies have been conducted extensively to elucidate its structure along with function^2,3^. This 459-amino acid transporter protein has 12 transmembrane domains (TMDs) with N- and C-termini located inside the cell^4,5^. It has two *N*-glycosylated Asn residues (Asn^58^ and Asn^68^), which are located in the first extracellular loop between TMDs 1 and 2^5^. The first intracellular loop (IL) between TMDs 2 and 3 has been suggested to be a functionally important reentrant loop having a β-turn structure formed by a DXXGRR sequence^6^, which begins at Asp^109^. In the loop, Asp^109^, Gly^112^, and Arg^113^ have been identified to be of particular importance^6–10^. For many of the TMDs, their roles in the structure and/or function of hPCFT have been identified, which include TMDs 3 and 6 that play the major role in its homo-oligomerization^11–13^, TMDs 2 and 4 that assist the oligomerization by the function of GXXXG motifs (G^93^XXXG^97^ in TMD2 and G^155^XXXG^159^ in TMD4)^11–13^, TMDs 2, 4, and 5 that are involved in substrate binding and translocation^6,12,14^, and TMDs 1, 2, 7, and 11 that forms an extracellular gate^15^. In addition, it has been suggested that TMDs 1, 3, 4, and 6 may potentially form a hydrophobic cavity involved in substrate binding^9,10^. Topologically, Gly^40^/Pro^41^ in TMD 1, Gly^158^/Gly^159^ in TMD 4, Pro^314^ in TMD 8, and Gly^361^/Tyr^362^/Gly^363^ in TMD 10 have been indicated to be potential break points, which are likely to play an important role in the conformational change^2^. Amino acid residues that are of particular importance have also been identified in TMDs, which include Glu^185^ in TMD 5 for proton coupling^16^, His^281^ in TMD 7 for proton and substrate binding^17^, Arg^376^ in TMD 10 for assisting proton and substrate binding^18^. Intracellularly located Ser^172^ (IL2) and His^247^ (IL3) have been suggested to be in interaction and together positioned to impact on hPCFT function^17^. There are also quite a few amino acid residues of which the mutations result in the impairments of hPCFT function linked to hereditary folate malabsorption^2^.

hPCFT has recently been found to be extensively inhibited by myricetin by a sustaining mechanism that persists after its removal^19–21^. The extensive and sustained inhibition of hPCFT by this flavonoid, which is widely present in vegetables and fruits^22^, raises a concern that it could potentially lead to harmful impairment of the intestinal absorption of those PCFT substrates. In beverages rich with myricetin, its concentration could be high enough, as typically reported to be 60 μM in red grape wine^22^, to induce the sustained inhibition, in which almost complete inhibition could be attained at its concentration of around 50 to 100 μM^21^. Thus, attention needs to be paid to the myricetin-induced hPCFT inhibition as one of potential drug interaction cases. Myricetin could be one of those flavonoids that act as perpetrators to inhibit enzymes or transporters involved in the disposition of drugs and nutrients^23–27^.

Although the sustaining mechanism of the myricetin-induced inhibition is yet to be fully clarified, kinetic analyses of the myricetin-induced inhibition of the uptake of folate in stably hPCFT-transfected Madin-Darby canine kidney II (MDCKII) cells and in the Caco-2 cell line, which is a well recognized intestinal epithelial cell model, consistently indicated that the maximum transport rate (V_max_) was reduced^19,20^. It is notable, however, that the Michaelis constant (K_m_) was reduced at the same time, attenuating the inhibition at lower folate concentrations. Since the expression of hPCFT, which was tagged with green fluorescent protein (GFP) for detection and stably introduced into MDCKII cells, was unchanged, being highly localized to the cellular membrane, it could be hypothesized that hPCFT undergoes some kind of myricetin-induced modulation to be less efficient for the translocation of folate bound with increased affinity. It should also be noted that the inhibition is of a sustained type, but can be reversed in a few hours^20^. Moreover, the inhibitory action of myricetin was suggested to be quite specific to hPCFT, as its orthologue in rat (rPCFT) and human riboflavin transporter 3 (hRFVT3/SLC52A3), which operates for the epithelial uptake of riboflavin (vitamin B2) in the intestine, were found not to be inhibited^21^.

Because of the myricetin-insensitive characteristic of rPCFT, rat cannot be used for investigation into the myricetin sensitivity of PCFT in detail, for example, by assessing its impact at the level of intestinal tissue or *in vivo*, even though the rat has been widely used as a model animal for such a purpose. However, the myricetin insensitivity of rPCFT can be taken advantage of to determine the amino acid residue involved in the myricetin-induced inhibition in hPCFT, as its myricetin sensitivity should be derived from the amino acid residues that are different from their counterparts in rPCFT. We here report our successful attempt to pursue that by analyses comparing between the two PCFTs. The acquired knowledge of the responsible amino acid residue would help advance the elucidation of the mechanism of the myricetin sensitivity of hPCFT and manage the potential risk of the myricetin-induced impairment of the intestinal absorption of folate and analogues.

## Results

### Myricetin sensitivity of chimeric constructs derived from hPCFT and rPCFT

**Figure 1:**
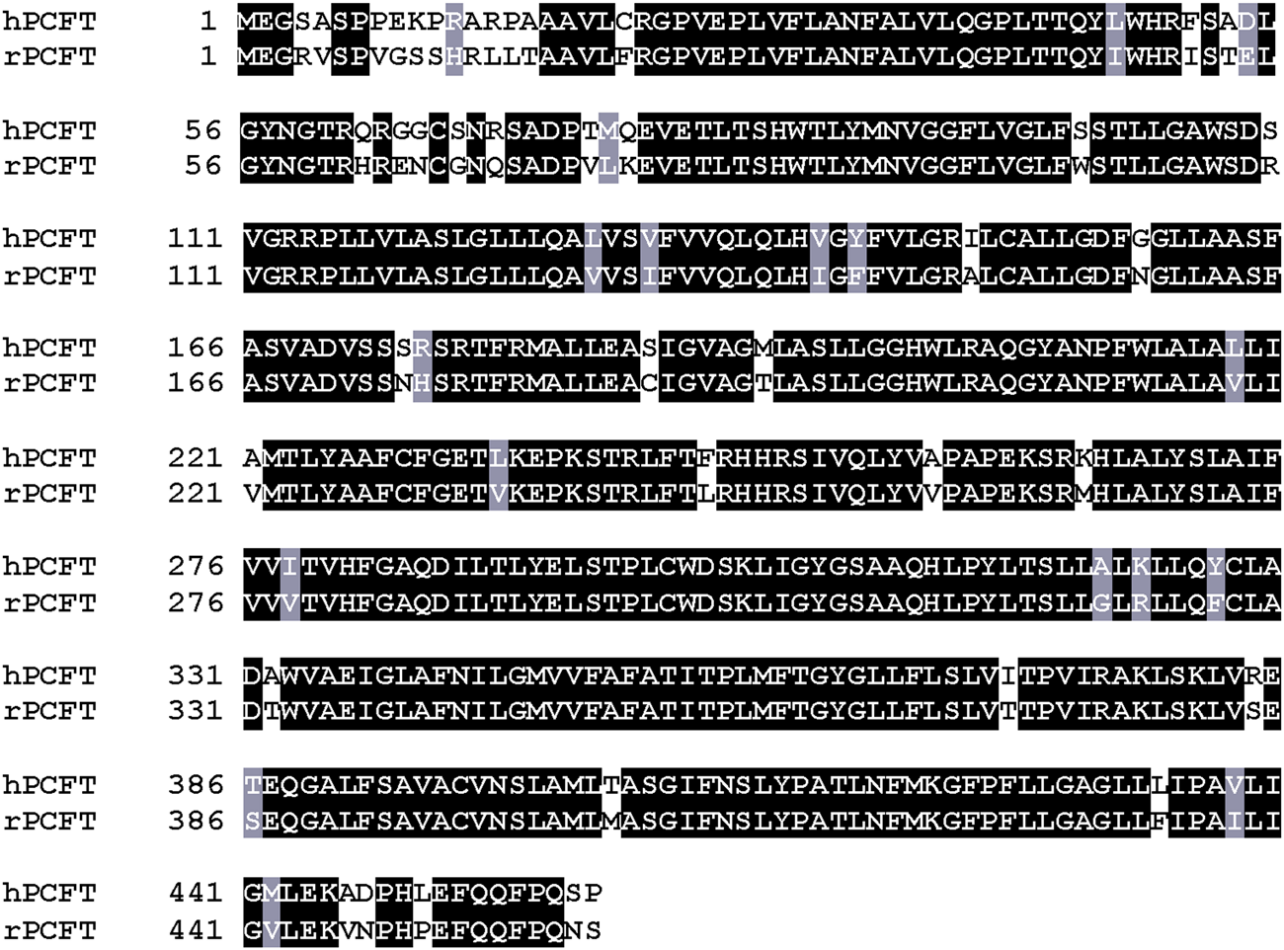
Alignment of the amino acid sequence of hPCFT with that of rPCFT. The amino acid sequence of hPCFT was aligned with that of rPCFT, using ClustalW program, and processed to visualize, using BOXSHADE program.

**Figure 2:**
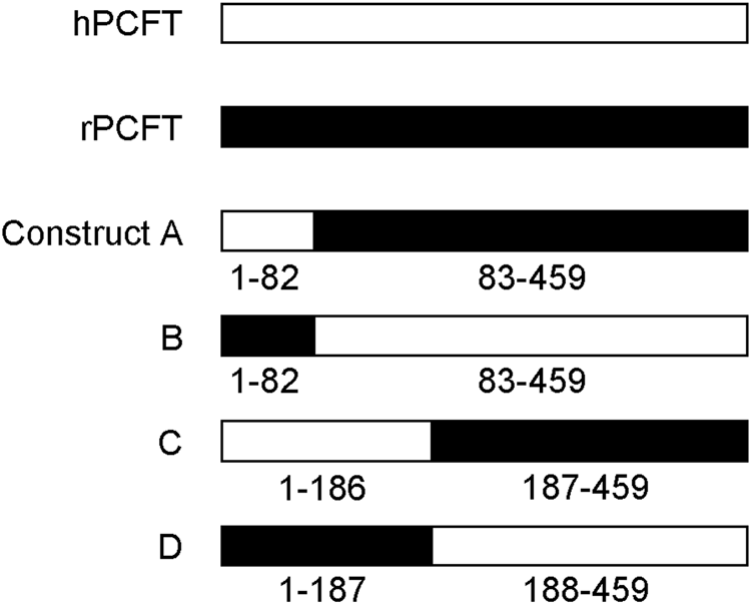
Schematic representation of chimeric constructs derived from hPCFT and rPCFT. The segments derived from hPCFT and rPCFT are indicated by bars in white and black, respectively, with the range of position numbers for amino acid residues.

hPCFT and rPCFT are highly analogous, both consisting of 459 amino acids, with 87% of them being identical^28^. The difference in sensitivity to myricetin between the two PCFTs should, therefore, be originated from the slight difference accounted for by the 13% of amino acids that are not identical (Fig. 1). It means, however, that there are still as many as 59 candidate amino acid residues that could be responsible for the myricetin sensitivity of hPCFT, being scattered throughout the molecule. Therefore, we first attempted to identify the hPCFT segment involved in the myricetin sensitivity to narrow down the candidate amino acid residues by a comparative analysis of the effects of myricetin on chimeric constructs derived from hPCFT and rPCFT (Fig. 2). Constructs A, B, C, and D have the 1st to 82nd, 83rd to 459th, 1st to 186th, and 188th to 459th amino acid residues, respectively, from hPCFT and those from rPCFT for the rest. Both of the constructs C and D have Cys derived from rPCFT at position 187 because of the procedure using the SphI restriction site to ligate the cDNAs derived from hPCFT and rPCFT, as described in the section of Methods.

To assess the sustained effect of myricetin with the minimal and essential kinetic characterization, the initial uptake of [^3^H]folate mediated by each chimeric construct was evaluated in transient transfectant human embryonic kidney 293 (HEK293) cells at the low and high folate concentrations of 5 nM and 5 μM, respectively, in the absence of myricetin after preincubation for 60 min in its presence at 100 μM. Those folate concentrations were previously determined to evaluate the approximate estimates of V_max_/K_m_, the transport coefficient in the linear phase of transport kinetics, and V_max_, respectively, for hPCFT and rPCFT, based on their K_m_ values of about 2 μM for folate^21,29,30^. Those estimates were used to assess myricetin-induced shifts in V_max_ and K_m_, where the latter could be estimated by dividing V_max_ by V_max_/K_m_. The uptake period was set to be 2 min, which was previously determined for the evaluation of the initial folate uptake by those PCFTs, and the medium condition was set to be pH 5.5, which was also determined previously for their optimal operation^21,29,30^.

**Figure 3:**
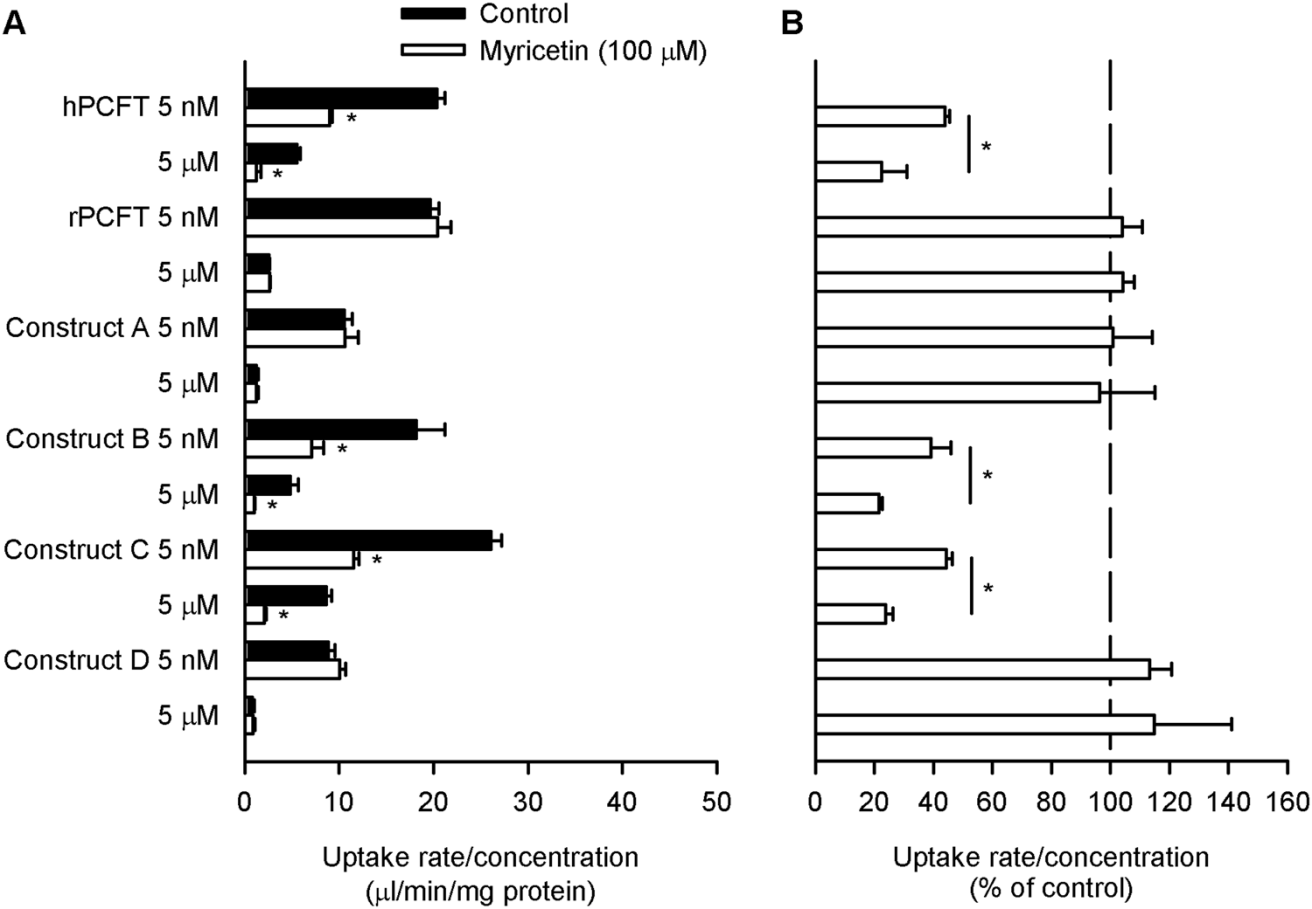
Effect of myricetin on the uptake of folate by chimeric PCFT constructs in transient transfectant HEK293 cells. The specific uptake of [^3^H]folate by each construct was evaluated at its concentrations of 5 nM and 5 µM for 2 min at pH 5.5 and 37 °C in the absence of myricetin after preincubation for 60 min in the presence of myricetin (100 µM), or in its absence (control), and subsequently for 5 min in its absence for washout. The data of uptake rate/concentration shown in Panel A are transformed to be presented in % of control for each in Panel B. The data for the wild type of hPCFT and rPCFT were cited for reference from a previous publication of our study conducted in conjunction with the present study^21^. Data are presented as the means ± S.E. (*n* = 4). In Panel A, for all the PCFTs and constructs, the uptake rate/concentration was significantly smaller at 5 µM than at 5 nM under the control condition (*p* < 0.05). *p < 0.05 com*p*ared with control for each (Panel A) or for comparison between the two concentrations for each PCFT or construct (Panel B).

For all the constructs, specific folate uptake was, when normalized to the concentration, smaller at the high concentration than at the low concentration in control cells without myricetin pretreatment (Fig. 3A), demonstrating the saturable characteristic of transport by a carrier-mediated mechanism, although their functional levels were varied. Among them, constructs B and C were sensitive to myricetin, as indicated by a reduction in specific folate uptake by myricetin, but constructs A and D were not (Fig. 3A,B). Moreover, for both of the myricetin-sensitive constructs of B and C, myricetin induced a greater extent of inhibition at the high folate concentration than at the low folate concentration (Fig. 3B), consistent with the characteristic of the myricetin-induced hPCFT inhibition described in our previous study in the aspect of apparent kinetics and indicating a reduction in both V_max_ and K_m_^19–21^. These results suggest that the amino acid residue responsible for the sensitivity of hPCFT to myricetin is present in the hPCFT-derived segment commonly included in the two constructs, which is the segment spanning 83rd to 186th amino acid residues. Accordingly, constructs A and D, in which the designated segment was derived from rPCFT, were insensitive to myricetin.

### Myricetin sensitivity of site-directed hPCFT mutants

Based on the analysis using the chimeric PCFT constructs, the candidate amino acid residues that could be responsible for the myricetin sensitivity of hPCFT were narrowed down to 10 of them that are different from their respective counterparts in rPCFT in the segment spanning 83rd to 186th amino acid residues (Fig. 1). We then prepared hPCFT mutants, in each of which one of the candidate amino acid residues was replaced with its counterpart in rPCFT by site-directed mutagenesis, and assessed the sustained effect of myricetin (100 μM) on their folate transport function in transient transfectant HEK293 cells.

**Figure 4:**
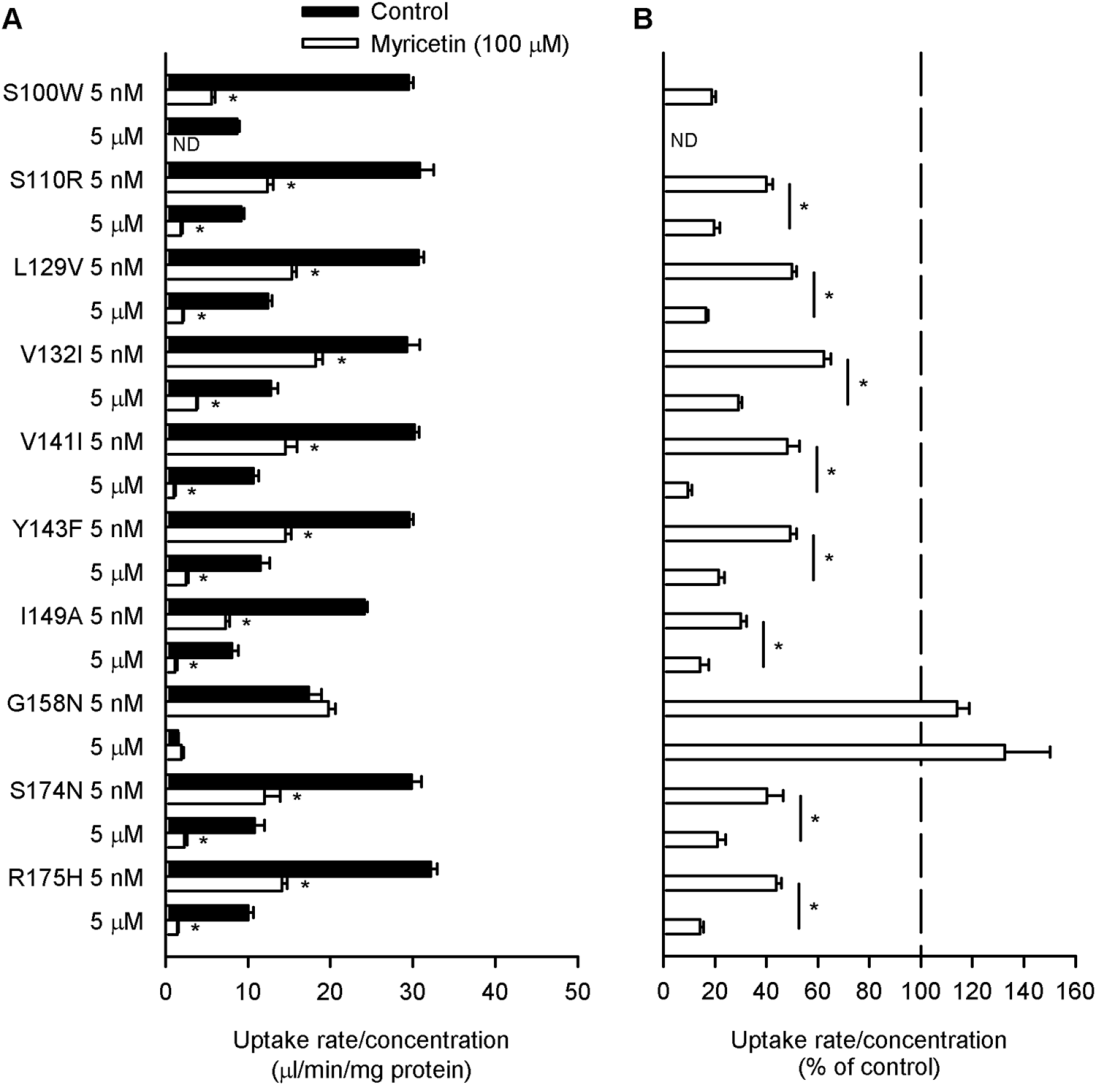
Effect of myricetin on the uptake of folate by various hPCFT mutants in transient transfectant HEK293 cells. The specific uptake of [^3^H]folate by each mutant was evaluated at its concentrations of 5 nM and 5 µM for 2 min at pH 5.5 and 37 °C in the absence of myricetin after preincubation for 60 min in the presence of myricetin (100 µM), or in its absence (control), and subsequently for 5 min in its absence for washout. The data of uptake rate/concentration shown in Panel A are transformed to be presented in % of control for each in Panel B. ND, not detected. Data are presented as the means ± S.E. (*n* = 4). In Panel A, for all the mutants, the uptake rate/concentration was significantly smaller at 5 µM than at 5 nM under the control condition (*p* < 0.05). *p < 0.05 com*p*ared with control for each (Panel A) or for comparison between the two concentrations for each mutant (Panel B).

**Figure 5:**
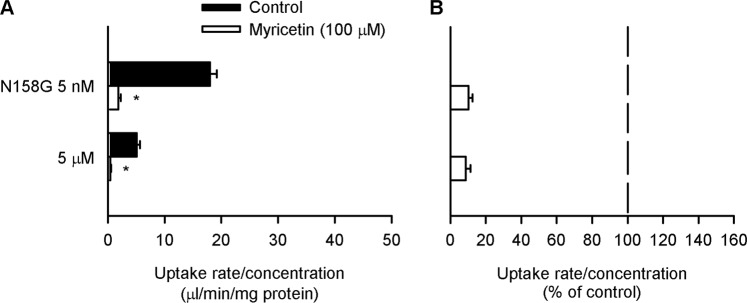
Effect of myricetin on the uptake of folate by the N158G mutant of rPCFT in transient transfectant HEK293 cells. The specific uptake of [^3^H]folate by the mutant was evaluated at its concentrations of 5 nM and 5 µM for 2 min at pH 5.5 and 37 °C in the absence of myricetin after preincubation for 60 min in the presence of myricetin (100 µM), or in its absence (control), and subsequently for 5 min in its absence for washout. The data of uptake rate/concentration shown in Panel A are transformed to be presented in % of control for each in Panel B. Data are presented as the means ± S.E. (*n* = 4). In Panel A, the uptake rate/concentration was significantly smaller at 5 µM than at 5 nM under the control condition (*p* < 0.05). *p < 0.05 com*p*ared with control for each (Panel A).

For all the mutants, specific folate uptake normalized to the concentration was smaller at the high concentration than at the low concentration in control cells without myricetin pretreatment (Fig. 4A), indicating that they all retain the saturable characteristic of transport by a carrier-mediated mechanism, although their functional levels were varied. Among them, only the G158N mutant was found to be insensitive to myricetin, without any alteration in its mediated folate uptake at both the low and high folate concentrations (Fig. 4A,B). All the other mutants were found to retain the myricetin sensitive characteristic similar to that observed for the wild type of hPCFT (Fig. 3) in the aspect of apparent kinetics^19–21^, their mediated folate uptake being reduced by myricetin to a greater extent at the high folate concentration than at the low folate concentration. These results suggest that Gly^158^ is the amino acid residue responsible for the myricetin sensitivity of hPCFT. Moreover, the opposite substitution of N158G transformed rPCFT, which is insensitive to myricetin, to be sensitive to myricetin, supporting the suggestion (Fig. 5). Although the characteristic of myricetin sensitivity of the N158G mutant was not fully in agreement with that of hPCFT, its mediated folate uptake being reduced to comparable extents at the low and high folate concentrations, the results indicate that Asn^158^ plays an important role in the myricetin insensitivity of rPCFT. In an additional set of experiments using the N158G mutant tagged with GFP for detection by western blotting and fluorescent microscopic observation, there was not any appreciable myricetin-induced alteration in the expression and localization of the mutant at the plasma membrane (Supplementary Figs. S1 and S2), suggesting that a decrease in V_max_ indicated by the reduced folate uptake at the high folate concentration implies a decrease in the velocity of substrate translocation, at least in agreement with the characteristic of myricetin-induced alteration in the V_max_ of hPCFT^19^. The saturable characteristic of transport by carrier-mediated mechanism was also confirmed for the N158G mutant (Fig. 5A).

**Figure 6:**
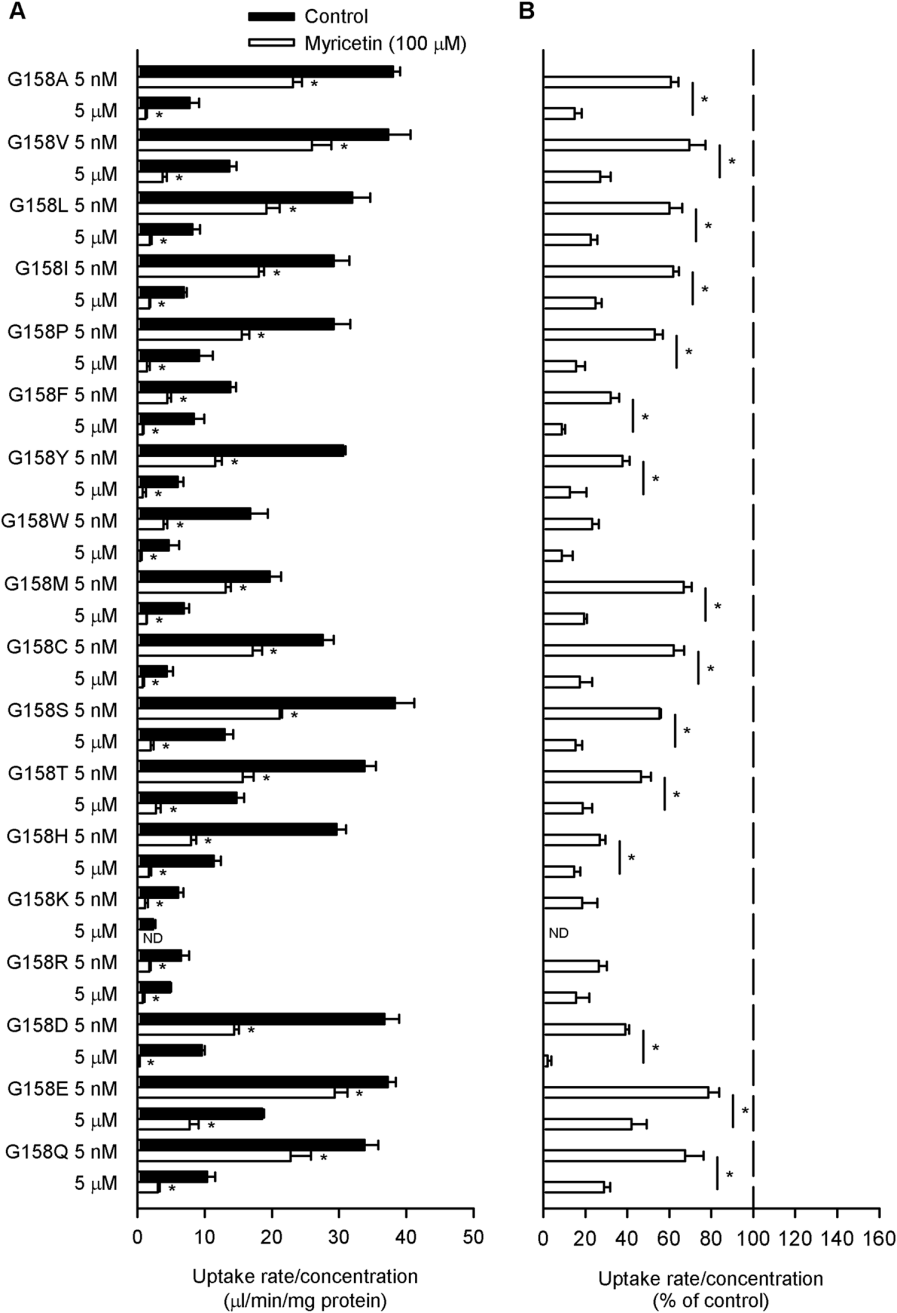
Effect of myricetin on the uptake of folate by various G158 mutants of hPCFT in transient transfectant HEK293 cells. The specific uptake of [^3^H]folate was evaluated at its concentrations of 5 nM and 5 µM for 2 min at pH 5.5 and 37 °C in the absence of myricetin after preincubation for 60 min in the presence of myricetin (100 µM), or in its absence (control), and subsequently for 5 min in its absence for washout. The data of uptake rate/concentration shown in Panel A are transformed to be presented in % of control for each in Panel B. ND, not detected. Data are presented as the means ± S.E. (*n* = 4). In Panel A, for all the mutants except for the G158R mutant, the uptake rate/concentration was significantly smaller at 5 µM than at 5 nM under the control condition (*p* < 0.05). *p < 0.05 com*p*ared with control for each (Panel A) or for comparison between the two concentrations for each mutant (Panel B).

We also prepared hPCFT mutants in which Gly^158^ was replaced with the other 18 amino acids that constitute human proteins and examined their myricetin sensitivity. The saturable characteristic of transport by a carrier-mediated mechanism was also retained in almost all of the mutants, with the only exception of the G158R mutant (Fig. 6A). Notably, for all the mutants, specific folate uptake was reduced by myricetin and, except for the G158W and G158R mutants, the reduction was greater in extent at the high folate concentration than at the low folate concentration (Fig. 6A,B). The results for all the mutants except for the G158W and G158R indicate a decrease in K_m_ as well as V_max_, suggesting that the myricetin-sensitive characteristic of hPCFT was retained in the aspect of apparent kinetics. In an additional set of experiments using G158 mutants tagged with GFP for detection, it was confirmed that none of them underwent any appreciable myricetin-induced alteration in the expression and localization at the plasma membrane (Supplementary Figs. S3 and S4) and, hence, it was suggested that a decrease in the velocity of substrate translocation was responsible for a decrease in the V_max_ indicated by the reduced folate uptake at the high folate concentration, as previously suggested for hPCFT^19^. Based on all these results, all the mutants except for G158W and G158R were suggested to fully retain the myricetin-sensitive characteristic of hPCFT. The G158W and G158R mutants were also at least sensitive to myricetin, similarly retaining the characteristic of myricetin-induced reduction in V_max_. Thus, the replacement of Gly^158^ with any amino acid other than Asn was found not to deprive of the myricetin-sensitive characteristic of hPCFT, although a slightly modulated type of inhibition was induced in the case of replacements with Trp and Arg. This finding suggests that Gly is not specifically required at the 158th position for myricetin to induce hPCFT inhibition, although the amino acid residue at the position should be critically important as a determinant for the myricetin sensitivity.

### Myricetin sensitivity of the PCFTs of some other selected animal species

**Figure 7:**
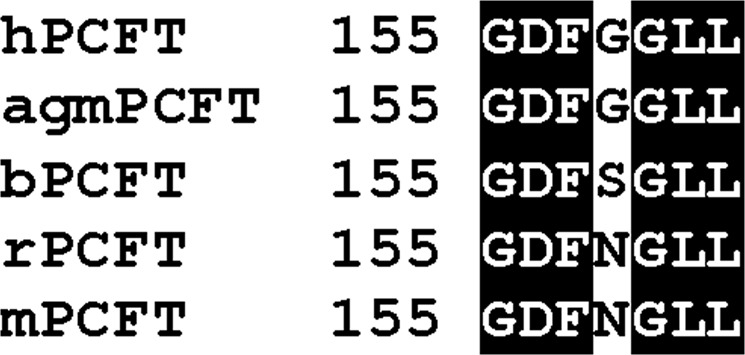
Alignment of the amino acid sequence of hPCFT with those of the PCFTs of selected animal species. The amino acid sequence of hPCFT at the vicinity of Gly^158^ was aligned with those of the designated PCFTs, using ClustalW program, and processed to visualize, using BOXSHADE program.

Assessing the myricetin sensitivity of the PCFTs of some other animals would be of help in further confirming the role of the amino acid residue at position 158 in that. We here selected the PCFTs of African green monkey (agmPCFT), bovine (bPCFT), and mouse (mPCFT), which have Gly, Ser, and Asn, respectively, at the position (Fig. 7), and assessed the sustained effect of myricetin (100 μM) on their folate transport function in transient transfectant HEK293 cells.

**Figure 8:**
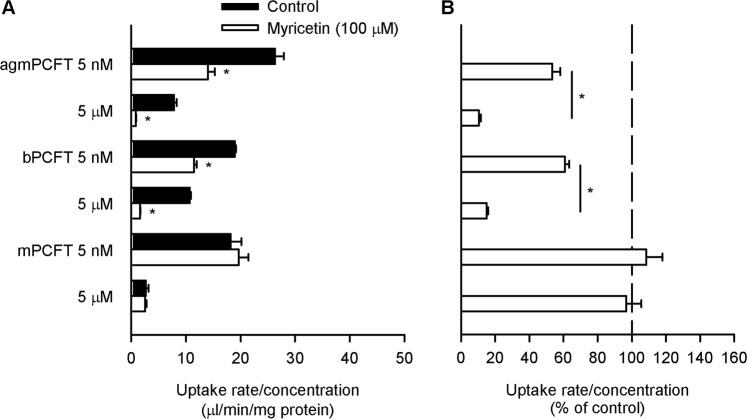
Effect of myricetin on the uptake of folate by the PCFTs of selected animal species in transient transfectant HEK293 cells. The specific uptake of [^3^H]folate was evaluated at its concentrations of 5 nM and 5 µM for 2 min at pH 5.5 and 37 °C in the absence of myricetin after preincubation for 60 min in the presence of myricetin (100 µM) or in its absence (control), and subsequently for 5 min in its absence for washout. The data of uptake rate/concentration shown in Panel A are transformed to be presented in % of control for each in Panel B. Data are presented as the means ± S.E. (*n* = 4). In Panel A, for all the PCFTs, the uptake rate/concentration was significantly smaller at 5 µM than at 5 nM under the control condition (*p* < 0.05). *p < 0.05 compared with control for each (Panel A) or for comparison between the two concentrations for each PCFT (Panel B).

As shown in Fig. 8A, for all the PCFTs, the saturable characteristic of transport by a carrier-mediated mechanism was confirmed. Among them, agmPCFT and bPCFT were found to be sensitive to myricetin, their mediated specific folate uptake being reduced by myricetin to a greater extent at the high folate concentration than at the low folate concentration, similarly to hPCFT (Fig. 8A,B). On the other hand, mPCFT, which has Asn at the position as rPCFT does, was found to be insensitive to myricetin, similarly to rPCFT. These results were consistent with those from the analysis using hPCFT mutants, supporting the suggested role of the amino acid residue at position 158 in the sensitivity of PCFTs to myricetin.

## Discussion

We have successfully identified Gly^158^ as an amino acid residue involved in the myricetin sensitivity of hPCFT in the present study. It is notable that the replacement of Gly^158^ in hPCFT with its counterpart of Asn in rPCFT, which is insensitive to myricetin, completely abolished its myricetin sensitivity and, oppositely, rPCFT acquired myricetin sensitivity when its Asn^158^ was replaced with Gly. This set of findings strongly suggests that Gly^158^ is the single amino acid residue responsible for the myricetin sensitivity in hPCFT. It is also notable that this amino acid residue, which is located in TMD 4 in hPCFT, has been indicated as one of those involved in the recognition of substrates, being located at the vicinity of the substrate binding pocket, and also in their subsequent translocation by conformational change^14^. More specifically, it has been predicted by topological analyses that Gly^158^ constitutes, together with neighbouring Gly^159^, a break point, which is among four of them present in hPCFT TMDs and presumed to play an important role in the conformational change^2,14^. The Gly^158^ could also play a role in the functionality of hPCFT through its involvement in the formation of hPCFT oligomers, since G^155^XXXG^159^ motif, in which Gly^158^ is located, has been suggested to be involved in, together with G^93^XXXG^97^ motif in TMD2, intramolecular packing and stability of transmembrane α helices and, thereby, assists homo-oligomerization of hPCFT at the interfaces formed by TMDs 3 and 6^11–13^. In addition, the importance of TMD4 in maintaining the integrity and/or functionality of hPCFT has been indicated by the instances of hPCFT defects due to the mutations of G147R and D156Y in TMD4, which were identified in patients of hereditary folate malabsorption^7,8^. Therefore, a direct modulation of Gly^158^ or a modulation of hPCFT that indirectly affects the residue could indeed be likely to induce a functional alteration of hPCFT. Gly is, however, not an amino acid that undergoes any typical modulation, being different from reactive amino acids that can undergo phosphorylation (Ser, Thr, and Tyr), glycosylation (Asn, Ser, and Thr) or disulphide-bond formation (Cys). Therefore, it may be more likely that an indirect mechanism, rather than a modulation of Gly^158^, is involved in the myricetin sensitivity of hPCFT. Consistent with this suggestion, the replacement of Gly^158^ with any amino acid other than Asn was found not to abolish the myricetin sensitivity of hPCFT, indicating that Gly is not the only amino acid specifically required at that position for the manifestation of the myricetin-induced inhibition. Alternatively, Asn may play a role in making PCFT resistant to the indirect effect caused by the myricetin-induced modulation. Interestingly, substituting Gln, which is highly analogous to Asn, for Gly did not cause any major alteration in the myricetin sensitivity of hPCFT, suggesting the involvement of a mechanism that delicately discriminates Asn from Gln. Since position 158 is located in a TMD, Asn is unlikely to undergo glycosylation at that position and, hence, its glycosylation status would not be a factor involved in the myricetin sensitivity. It would not be involved in that even if Asn might be glycosylated at that position, since the myricetin-insensitive characteristic of rPCFT, which has Asn^158^, was retained after deglycosylation treatment using tunicamycin (Supplementary Table S4). The finding suggests that deglycosylation at Asn^158^ has no impact on that characteristic of rPCFT, if it might be glycosylated, or the residue is not glycosylated. The treatment was, on the other hand, suggested to be effective in deglycosylating rPCFT, as its mediated folate uptake in cells without myricetin pretreatment was, as assessed at the low folate concentration (5 nM), significantly greater in cells untreated with tunicamycin than in those treated, which indicates the suppression of the transport function of rPCFT by tunicamycin treatment presumably by deglycosylation at Asn^58^ and Asn^68^ in the first extracellular loop, similarly to the earlier finding for hPCFT^4^. There may be a possibility that a series of events triggered by myricetin is involved in the inhibition, as suggested from our previous finding that a similar type of inhibition was induced by wortmannin, an inhibitor of phosphoinositide 3-kinases (PI3Ks)^20^. Myricetin might act on PI3Ks or relevant signalling pathway to induce a series of events that leads to the hPCFT inhibition. It was, however, suggested at the same time that the contribution of the pathway related to PI3Ks could be only partial. Although further studies are needed to elucidate the underlying mechanism, it is at least undoubtful that the amino acid residue at position 158 should be responsible for the animal species difference in the myricetin sensitivity of PCFT.

The myricetin-sensitive characteristic seems to have been rendered to PCFTs in a certain class of higher animals by mutation at position 158 in the evolutionary process. Since Asn encoded by AAC, as in rPCFT and mPCFT as myricetin-insensitive ones, cannot be replaced with Gly encoded by GGT, as in hPCFT and agmPCFT as myricetin-sensitive ones, or Ser encoded by AGT, as in bPCFT as another myricetin-sensitive one, by a single nucleotide substitution, the amino acid substitution from Asn to Gly or Ser in those cases may have occurred as a consequence of multiple steps of single nucleotide substitution. The myricetin-sensitive types of PCFTs may have been maintained because of their advantage in some biological aspect. It could be related to some biological function of PCFT or folate that would be benefited by being restricted in the presence of myricetin, which is abundant in various fruits and vegetables. The possibility cannot be ruled out or may be rather likely that myricetin ingested regularly from such dietary plant sources could not be so concentrated as in myricetin-rich beverages, but there might be some other analogous flavonoids or compounds that act on hPCFT to induce the sustained inhibition by the same mechanism more potently or in an additive manner. The specific role of the myricetin sensitivity is, thus, unknown at this time and awaits to be explored in the future. Nevertheless, caution would need to be taken for the excessive ingestion of myricetin-rich beverages to avoid the potential risk of the malabsorption of folate and analogues.

In conclusion, we have successfully identified Gly^158^ as the amino acid residue responsible for the myricetin sensitivity of hPCFT. This finding provides a molecular basis for the specific action of myricetin on hPCFT. It should also be of help in guiding future studies to investigate further into the mechanism of the inhibition and manage the potential risk arising from that.

## Methods

### Cells and culture

The provider of HEK293 cells and COS-7 cells was Cell Resource Center for Biomedical Research, Tohoku University, Japan, and that of Madin-Darby bovine kidney (MDBK) cells and RAW264.7 cells was RIKEN BioResource Research Center (Tsukuba, Japan). The cells were maintained at 37 °C and 5% CO_2_ in a growth medium supplemented with 10% fetal bovine serum (Sigma-Aldrich, St. Louis, MO, USA) and 1% penicillin/streptomycin, as described previously^19–21^. The growth medium was RPMI 1640 medium (Wako Pure Chemical Industries, Osaka, Japan) for RAW264.7 cells and Dulbecco’s modified Eagle’s medium (Wako Pure Chemical Industries) for the others.

### Plasmids

For the generation of the cDNAs for chimeric constructs and mutants of PCFTs, the cDNAs of hPCFT and rPCFT, which were prepared previously^29,30^, were used as templates. They were all incorporated in pCI-neo vector (Promega, Madison, WI, USA). The GenBank accession numbers for the cDNAs of hPCFT and rPCFT are NM_080669.4 and NM_001013969.1, respectively.

For the generation of the cDNAs for constructs A and B, cDNAs for the specified segments derived from hPCFT and rPCFT were amplified by PCR, using the primers designed to incorporate an SpeI restriction site (Supplementary Table S1) and KOD-plus-neo as a polymerase (Toyobo, Osaka, Japan), and ligated at the SpeI site. The cDNAs for constructs C and D were similarly generated, where the primers for hPCFT were designed to incorporate an SphI restriction site and those for rPCFT were designed to utilize an SphI restriction site present in its cDNA sequence for the 187th amino acid residue of Cys (Supplementary Table S1).

The cDNAs for the mutants, in each of which a designated single amino acid residue was replaced in hPCFT or rPCFT, were generated by a site-directed mutagenesis method (PrimeSTAR Mutagenesis Basal Kit, Takara Bio, Kusatsu, Japan). The primers for PCR are shown in Supplementary Table S2.

The cDNA of agmPCFT (GenBank accession number, XM_008010782.1) was cloned by a reverse transcription (RT)-polymerase chain reaction (PCR) method, as described previsouly^31^. In brief, an RT reaction was carried out to obtain cDNA mixture from total RNA prepared from COS-7 cells by a guanidine isothiocyanate extraction method^32^, using 1 μg of the total RNA, an oligo(dT) primer, and ReverTra Ace (Toyobo) as a reverse transcriptase. The cDNA of agmPCFT was amplified by PCR, using PrimeSTAR Max DNA Polymerase (Takara Bio). Then, the second PCR was performed using the amplified product as a template to incorporate an EcoRI restriction site. Similarly, the cDNAs of bPCFT and mPCFT were cloned from MDBK cells and RAW264.7 cells, respectively. Their GenBank accession numbers are NM_001079585.1 and NM_026740.2, respectively. The primers for PCR are shown in Supplementary Table S3.

All the final cDNA products were incorporated into pCI-neo vector to prepare plasmids for transfection and their sequences were determined with an automated sequencer (ABI PRISM 3130; Applied Biosystems, Foster City, CA, USA), as described previously^31^.

### Uptake assays

HEK293 cells (2.0 × 10^5^ cells/ml, 1 ml/well) were grown on 24-well plates coated with poly-L-lysine for 12 h, transfected with the plasmid carrying the cDNA of the designated transporter, chimeric construct, or mutant, using Lipofectamine 2000 (Invitrogen, Carlsbad, CA, USA), and cultured for 48 h for transient expression, as described previously^21^.

Uptake assays were conducted as described previously^20,21^, using a modified Hanks’ solution supplemented with 10 mM 2-(*N*-morpholino) ethanesulfonic acid (pH 5.5). In brief, the cells in each well were preincubated for 60 min in 1 ml of the modified Hanks’ solution with or without myricetin (100 μM) and subsequently, after replacing the solution with fresh one without myricetin, preincubated for 5 min for washout. Then the solution was replaced with 0.25 ml of the modified Hanks’ solution added with [^3^H]folate (Moravek, Brea, CA, USA) to start uptake in the absence of myricetin. All the procedures were conducted at 37 °C. After uptake assays were stopped by addition of ice-cold modified Hanks’ solution (2 ml), the cells were washed with the same solution and, then, solubilized to determine the associated radioactivity by liquid scintillation counting for the evaluation of folate uptake. The specific uptake of folate was estimated by subtracting its uptake in mock cells from that in transfectant cells. The uptake was normalized to cellular protein content, which was determined by the bicinchoninic acid (BCA) method (BCA Protein Assay Reagent Kit; Thermo Fisher Scientific, Waltham, MA, USA), using bovine serum albumin as the standard.

### Statistical analysis

Student’s *t*-test was used for statistical analysis, where the difference in means was considered significant when the *p*-value was less than 0.05.

## Supplementary information

Supplementary information
