# Supplementary material for: Identification of the amino acid residue responsible for the myricetin sensitivity of human proton-coupled folate transporter
Source: Sci Rep. 2019 Dec 2;9:18105. doi: 10.1038/s41598-019-54367-9 (PMC6889420; doi:10.1038/s41598-019-54367-9)
Supplement: Supplementary file 1 — Supplementary information [file 41598_2019_54367_MOESM1_ESM.pdf]

Supplementary Information

Title: Identification of the amino acid residue responsible for the myricetin sensitivity of human proton-coupled folate transporter

Authors: Takahiro Yamashiro, Tomoya Yasujima, Kinya Ohta, Katsuhisa Inoue & Hiroaki Yuasa

Supplementary Methods

Plasmids for GFP-tagged transporters. The plasmids for the designated transporters tagged with GFP were prepared by transferring their cDNAs into pEGFP-C1 vector (Clontech, MountainView, CA), as described previously[29].

Western blot analysis. Western blot analysis was conducted to examine the protein expression of the designated transporters in the plasma membrane in transient transfectant HEK293 cells, using those tagged with GFP for detection, as described previously[19]. The primary antibody was mouse anti-GFP (Proteintech, Rosemont, IL, USA) and used at a dilution of 1:1,000. The secondary antibody was goat anti-mouse IgG conjugated to horseradish peroxidase (Sigma-Aldrich) and used at a dilution of 1:10,000. For the detection of β-actin as a loading control, mouse anti-β-actin (Sigma-Aldrich) was used as the primary antibody at a dilution of 1:1,000.

Fluorescent microscopic analysis. Fluorescent microscopic analysis was conducted to examine the cellular localization of the designated transporters in transient transfectant HEK293 cells, using those tagged with GFP and visualizing GFP-derived fluorescence with a confocal laser-scanning microscope (LSM510, Carl Zeiss, Jena, Germany), as described previously[19].

Table S1. Primers for Amplification of the cDNAs for the Specified Segments Derived from hPCFT and rPCFT for the Generation of Chimeric Constructs

Construct	Segment	Orientation	Sequence (5' to 3')
A	hPCFT (1-82)	Forward	CTCTCCACAGGTGTCCACTC
		Reverse	GTGACTAGTAAGGGTCTCCACTT
	rPCFT (83-459)	Forward	CTGACTAGTCACTGGACCCTCTA
		Reverse	TTCACTGCATTCTAGTTGTGG
B	rPCFT (1-82)	Forward	CTCTCCACAGGTGTCCACTC
		Reverse	GTGACTAGTCAGGGTCTCCACTT
	hPCFT (83-459)	Forward	CTTACTAGTCACTGGACCCTCTA
		Reverse	TTCACTGCATTCTAGTTGTGG
C	hPCFT (1-186)	Forward	CTCTCCACAGGTGTCCACTC
		Reverse	GATGCATGCTTCCAGCAGGGCCAT
	rPCFT (187-459)	Forward	GAAGCATGCATTGGTGTGGCC
		Reverse	TTCACTGCATTCTAGTTGTGG
D	rPCFT (1-186)	Forward	CTCTCCACAGGTGTCCACTC
		Reverse	AATGCATGCTTCCAGCAGAGCCAT
	hPCFT (187-459)	Forward	GAAGCATGCATCGGGGTGGCT
		Reverse	TTCACTGCATTCTAGTTGTGG

Underlined with solid and broken lines are restriction sites for SpeI and SphI, respectively. Segments are specified by the range of position numbers for amino acid residues in parentheses.

Table S2. Primers for Generation of the cDNAs for PCFT Mutants

PCFT	Mutant[a]	Orientation	Sequence (5' to 3')
hPCFT	S100W (TCG → TGG)	Forward	TGGTCCACCCTGCTGGGAGCTT
		Reverse	GAAGAGCCCCACCAGGAA
	S110R (AGT → CGC)	Forward	AGCGACCGCGTGGGCCGCCGCCCGCTG
		Reverse	GCCCACGCGGTCGCTCCAAGCTCCCAG
	L129V (CTA → GTG)	Forward	GTGGTGTCCGTTTTTGTGGTGCA
		Reverse	GGCCTGGAGCAGCAGGCCCA
	V132I (GTT → ATC)	Forward	GTGTCCATCTTTGTGGTGCAGCTGCAG
		Reverse	CACAAAGATGGACACTAGGGCCTGGAG
	V141I (GTC → ATC)	Forward	ATCGGCTACTTCGTGCTGGGT
		Reverse	GTGGAGCTGCAGCTGCACCA
	Y143F (TAC → TTC)	Forward	GTCGGCTTCTTCGTGCTGGGTCGCATC
		Reverse	CACGAAGAAGCCGACGTGGAGCTGCAG
	I149A (ATC → GCC)	Forward	GCCCTTTGTGCCCTCCTCGG
		Reverse	GCGACCCAGCACGAAGTA
	G158N (GGT → AAC)	Forward	GACTTCAACGGCCTTCTGGCTGCTAGC
		Reverse	AAGGCCGTTGAAGTCGCCGAGGAGGGC
	G158A (GGT → GCC)	Forward	GACTTCGCCGGCCTTCTGGCTGCTAGC
		Reverse	AAGGCCGGCGAAGTCGCCGAGGAGGGC
	G158V (GGT → GTG)	Forward	GACTTCGTGGGCCTTCTGGCTGCTAGC
		Reverse	AAGGCCCACGAAGTCGCCGAGGAGGGC
	G158L (GGT → CTG)	Forward	GACTTCCTGGGCCTTCTGGCTGCTAGC
		Reverse	AAGGCCCAGGAAGTCGCCGAGGAGGGC
	G158I (GGT → ATC)	Forward	GACTTCATCGGCCTTCTGGCTGCTAGC
		Reverse	AAGGCCGATGAAGTCGCCGAGGAGGGC
	G158P (GGT → CCC)	Forward	GACTTCCCCGGCCTTCTGGCTGCTAGC
		Reverse	AAGGCCGGGGAAGTCGCCGAGGAGGGC
	G158F (GGT → TTC)	Forward	GACTTCTTCGGCCTTCTGGCTGCTAGC
		Reverse	AAGGCCGAAGAAGTCGCCGAGGAGGGC
	G158Y (GGT → TAC)	Forward	GACTTCTACGGCCTTCTGGCTGCTAGC
		Reverse	AAGGCCGTAGAAGTCGCCGAGGAGGGC

To be continued. [a] Substitution of the nucleotide sequence for the mutation of the designated amino acid is indicated in parentheses.

Table S2. Continued.

PCFT	Mutant[a]	Orientation	Sequence (5' to 3')
	G158W (GGT → TGG)	Forward	GACTTCTGGGGCCTTCTGGCTGCTAGC
		Reverse	AAGGCCCCAGAAGTCGCCGAGGAGGGC
	G158M (GGT → ATG)	Forward	GACTTCATGGGCCTTCTGGCTGCTAGC
		Reverse	AAGGCCCATGAAGTCGCCGAGGAGGGC
	G158C (GGT → TGC)	Forward	GACTTCTGCGGCCTTCTGGCTGCTAGC
		Reverse	AAGGCCGCAGAAGTCGCCGAGGAGGGC
	G158S (GGT → AGT)	Forward	GACTTCAGTGGCCTTCTGGCTGCTAGC
		Reverse	AAGGCCACTGAAGTCGCCGAGGAGGGC
	G158T (GGT → ACC)	Forward	GACTTCACCGGCCTTCTGGCTGCTAGC
		Reverse	AAGGCCGGTGAAGTCGCCGAGGAGGGC
	G158H (GGT → CAC)	Forward	GACTTCCACGGCCTTCTGGCTGCTAGC
		Reverse	AAGGCCGTGGAAGTCGCCGAGGAGGGC
	G158K (GGT → AAG)	Forward	GACTTCAAGGGCCTTCTGGCTGCTAGC
		Reverse	AAGGCCCTTGAAGTCGCCGAGGAGGGC
	G158R (GGT → AGA)	Forward	GACTTCAGAGGCCTTCTGGCTGCTAGC
		Reverse	AAGGCCTCTGAAGTCGCCGAGGAGGGC
	G158D (GGT → GAC)	Forward	GACTTCGACGGCCTTCTGGCTGCTAGC
		Reverse	AAGGCCGTCGAAGTCGCCGAGGAGGGC
	G158E (GGT → GAG)	Forward	GACTTCGAGGGCCTTCTGGCTGCTAGC
		Reverse	AAGGCCCTCGAAGTCGCCGAGGAGGGC
	G158Q (GGT → CAG)	Forward	GACTTCCAGGGCCTTCTGGCTGCTAGC
		Reverse	AAGGCCCTGGAAGTCGCCGAGGAGGGC
	S174N (AGT → AAT)	Forward	AGCTCCAATCGCAGCCGCACCTTCCGG
		Reverse	GCTGCGATTGGAGCTGACATCTGCCAC
	R175H (CGC → CAC)	Forward	TCCAGTCACAGCCGCACCTTCCGGATG
		Reverse	GCGGCTGTGACTGGAGCTGACATCTGC
rPCFT	N158G (AAC → GGT)	Forward	GATTTCGGTGGCCTTCTTGCTGCTAGC
		Reverse	AAGGCCACCGAAATCTCCCAGAAGGGC

[a] Substitution of the nucleotide sequence for the mutation of the designated amino acid is indicated in parentheses.

Table S3. Primers for Amplification of the cDNAs of amgPCFT, bPCFT, and mPCFT

PCFT	PCR	Orientation	Sequence (5' to 3')
amgPCFT	1st	Forward	CAGTCCCAGGCAGCCAGTCG
		Reverse	AAGGAAGAACTGACTCCTGTCCCGA
	2nd	Forward	GCTGAATTCAGACATGGAGGGGAGCGCG
		Reverse	AAGGAAGAACTGACTCCTGTCCCGA
bPCFT	1st	Forward	CTGCAAGAAACCCGAAAGCA
		Reverse	ACTGACTTATGATGATTGGCACT
	2nd	Forward	AAGCTCGAGAGCATGGAGGGACGCGCGAA
		Reverse	TCAGCGGCCGCTCAGGGGCTCTGGGAAAAC
mPCFT	1st	Forward	CCTCCCGCAGCTGGTTCCGAG
		Reverse	TGCTGGGCTTTAGGCATACATCGTC
	2nd	Forward	CCGAATTCGAGCATGGAGGGGCGCGT
		Reverse	TCCTCATGGCTTCTAGAAGATCAGGG

Underlined with solid, broken, dotted, and double lines are restriction sites for EcoRI, XhoI, NotI, and XbaI, respectively.

Table S4. Myricetin Insensitivity of rPCFT in Transient Transfectant HEK293 Cells Pretreated with Tunicamycin

Pretreatment		Folate concentration	Uptake rate/concentration
Tunicamycin	Myricetin		(μl/min/mg protein)
-	-	5 nM	12.68 ± 0.30*
+	-	5 nM	9.01 ± 0.27
+	+	5 nM	9.52 ± 0.26
+	-	5 μM	0.54 ± 0.22
+	+	5 μM	0.71 ± 0.12

The specific uptake of [^{3}H]folate by rPCFT was evaluated at its concentrations of 5 nM and 5 μM for 2 min at pH 5.5 and 37 ºC in the absence of myricetin after preincubation for 60 min in the presence (+) of myricetin (100 μM), or in its absence (-), and subsequently for 5 min in its absence for washout. The experiments were conducted using cells cultured in the presence (+) of tunicamycin (1 μg/ml), or in its absence (-), for the 48 h-period for the transient expression of rPCFT. Data are presented as the means ± S.E. (n = 4). *, $p < 0.05$ compared with the value for 5 nM folate in cells pretreated with tunicamycin but not with myricetin.

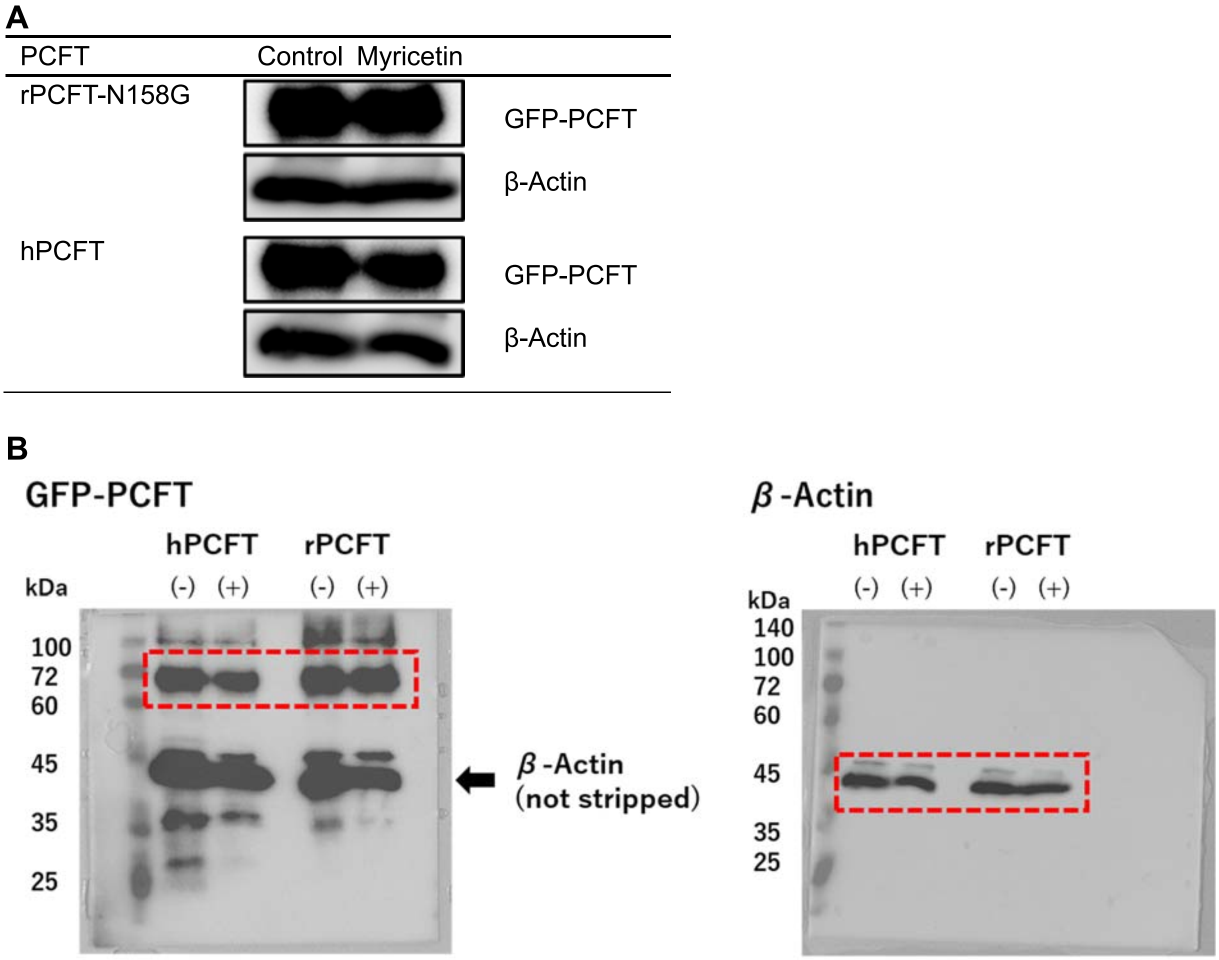

Figure S1. Effect of myricetin on the expression of the GFP-tagged N158G mutant of rPCFT at the plasma membrane of transient transfectant HEK293 cells. The cells were pretreated for 60 min without (control) or with myricetin (100 μM). Western blot analysis was conducted by probing for GFP tagged to the mutant in the crude membrane fraction (20 μg protein aliquots). The blots of β-actin are shown for reference. The blots of hPCFT (wild type) are also shown for reference. The blots in Panel A were cropped from the original images shown in Panel B, in which the blots of GFP-PCFTs and β-actin are framed by broken lines and the blots for control and myricetin-treated cells are indicated by - and +, respectively.

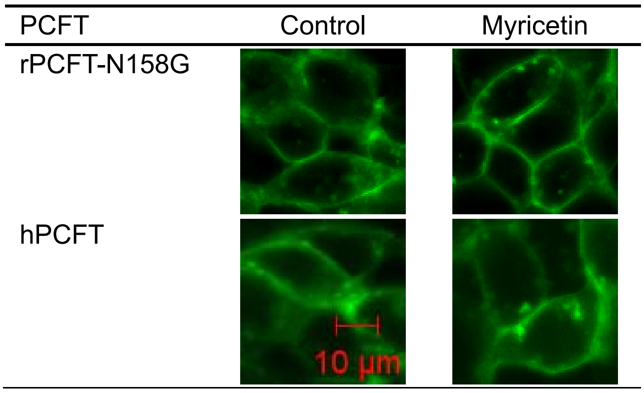

Figure S2. Effect of myricetin on the cellular localization of the GFP-tagged N158G mutant of rPCFT in transient transfectant HEK293 cells. GFP-derived green fluorescence is mostly and similarly localized at the plasma membrane, outlining the cells, in those pretreated for 60 min without (control) or with myricetin (100 µM). The images for hPCFT (wild type) are shown for reference. All the images are at the same magnification represented by the scale bar in the control image for hPCFT.

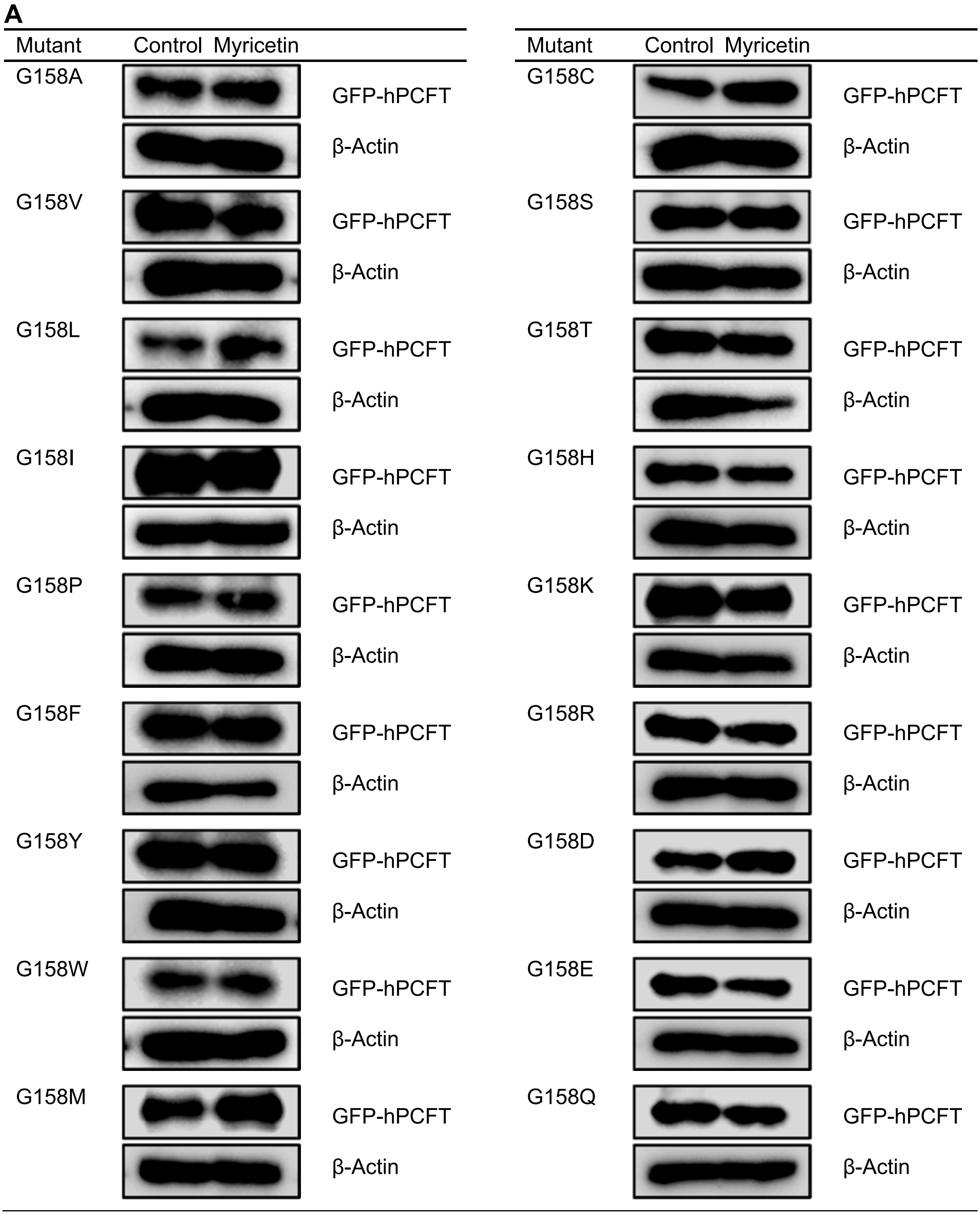

Figure S3. Effect of myricetin on the expression of the GFP-tagged G158 mutants of hPCFT at the plasma membrane of transient transfectant HEK293 cells. The cells were pretreated for 60 min without (control) or with myricetin (100 μM). Western blot analysis was conducted by probing for GFP tagged to the mutants in the crude membrane fraction (20 μg protein aliquots). The blots of β-actin are shown for reference. The blots in Panel A were cropped from the original images shown in Panel B, in which the blots of GFP-hPCFT mutants and β-actin are framed by broken lines and the blots for control and myricetin-treated cells are indicated by - and +, respectively. To be continued.

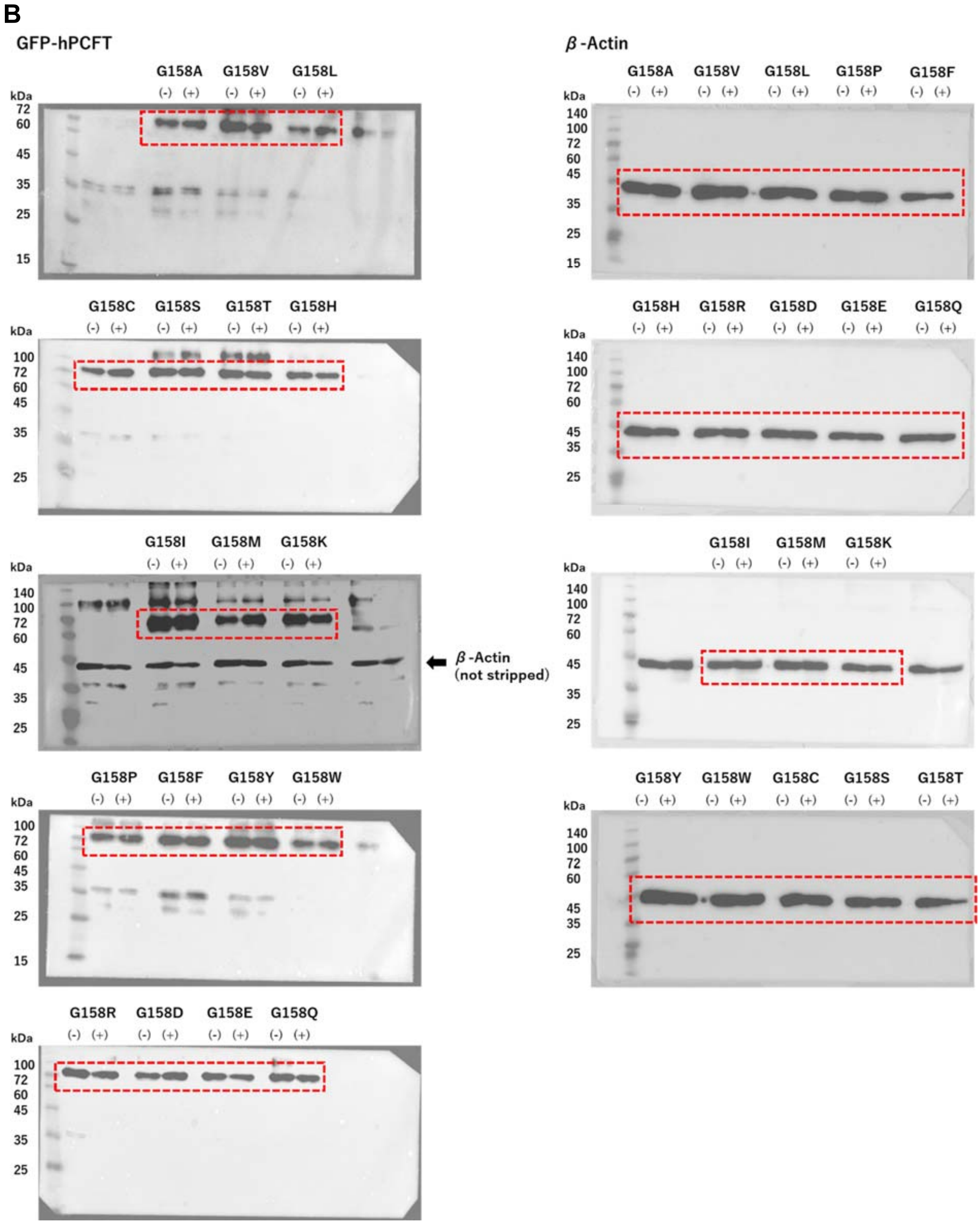

Figure S3. Continued.

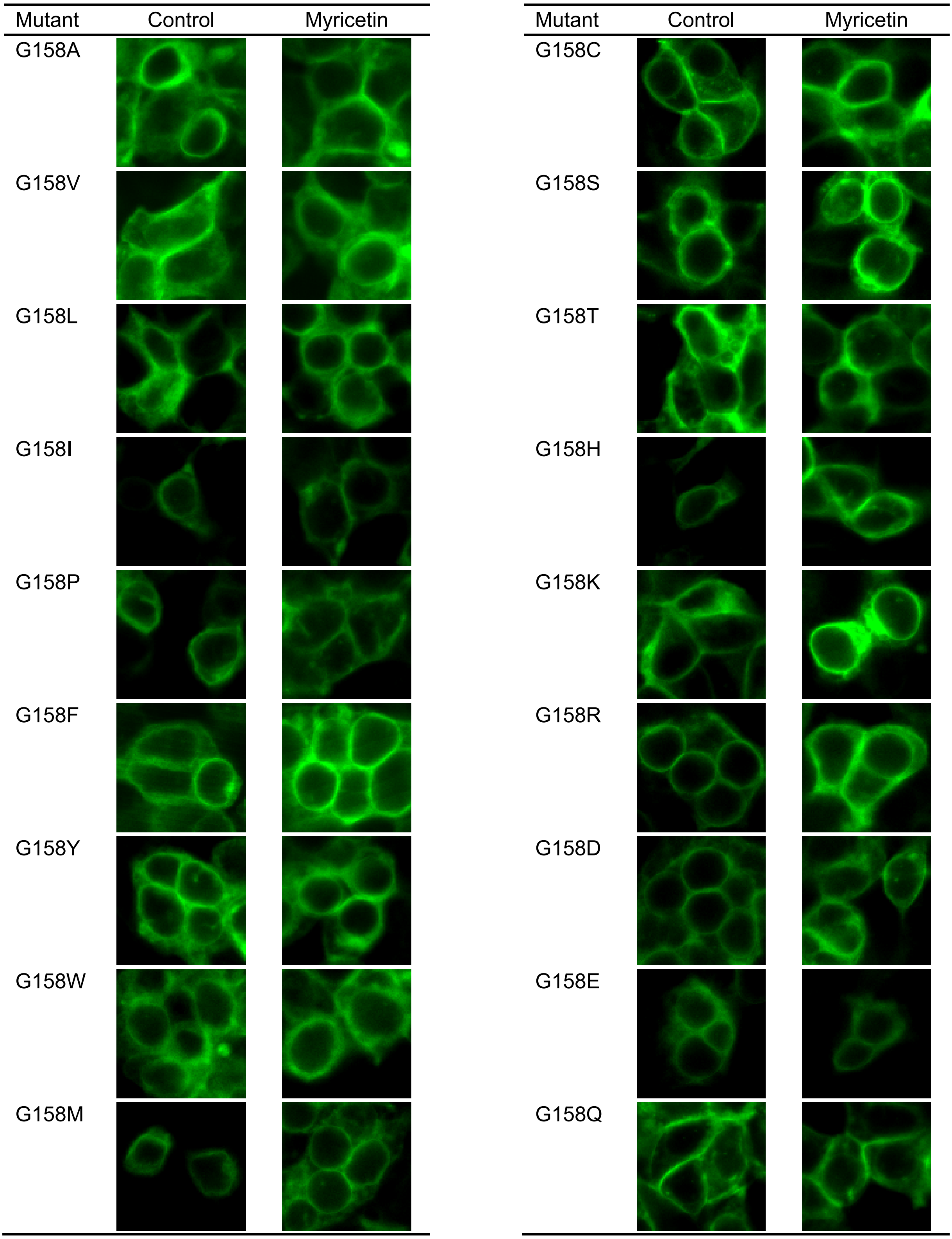

Figure S4. Effect of myricetin on the cellular localization of the GFP-tagged G158 mutants of hPCFT in transient transfectant HEK293 cells. GFP-derived green fluorescence is mostly and similarly localized at the plasma membrane, outlining the cells, in those pretreated for 60 min without (control) or with myricetin (100 μM). All the images are at the same magnification represented by the scale bar in the control image for hPCFT in Fig. S2.
